# Higher serum choline and betaine levels are associated with better body composition in male but not female population

**DOI:** 10.1371/journal.pone.0193114

**Published:** 2018-02-20

**Authors:** Xiang Gao, Edward Randell, Haicheng Zhou, Guang Sun

**Affiliations:** 1 College of Life Sciences, Qingdao University, Qingdao, Shandong, China; 2 Faculty of Medicine, Memorial University, St. John’s, Newfoundland and Labrador, Canada; 3 College of Food Science and Engineering, Ocean University of China, Qingdao, Shandong Province, China; 4 The Department of Endocrinology, the First Affiliated Hospital of Dalian Medical University, Dalian, Liaoning, China; Nathan S Kline Institute, UNITED STATES

## Abstract

**Background:**

Animal studies proved that choline and betaine have beneficial effect on reducing body fat. However, evidence in humans is scarce. We aim to investigate the association between serum choline and betaine levels with body composition in general population.

**Methods:**

This is an observational cross-sectional study performed in 1081 subjects from the CODING (Complex Disease in Newfoundland population: Environment and Genetics) study. Serum choline and betaine levels were measured based on liquid chromatography coupled with tandem mass spectrometry technology. Body composition was measured using dual-energy X-ray absorptiometry following a 12-hour fast. Major confounding factors including age, sex, total calorie intake and physical activity level were controlled in all analyses.

**Results:**

Significantly inverse correlations were found between serum betaine levels and all obesity measurements in males (r ranged from -0.12 to -0.23, and p<0.01 for all) but not in females. Serum choline was negatively associated with total percent body fat (%BF), percent trunk fat (%TF), weight, body mass index (BMI), waist circumference (WC), and waist-to-hip ratio (r ranged from -0.11 to -0.19, and p<0.05 for all) in males and positively associated with weight, BMI and WC (r ranged from 0.09 to 0.10, and p<0.05 for all) in females. The negative associations between serum choline and betaine levels with obesity in males were more profound in those not on any medication than those taking medications. Moreover, obese males had the lowest serum choline and betaine levels, followed by overweight males, and normal weight males having the highest serum choline and betaine levels, especially in those not taking medications (p<0.05). Likewise, subjects with the highest serum levels of both had the lowest obesity indexes, especially those not taking medications.

**Conclusions:**

Higher serum choline and betaine levels were associated with a more favorable body composition (lower body fat and higher lean body mass) in males and the favorable association was more pronounced in non-medication users.

## Introduction

Choline and betaine are metabolically related quaternary ammonium compounds [1]. Choline is recognized as an essential nutrient for maintaining human health and a precursor to neurotransmitter acetylcholine, to cell membrane phospholipids and lipoproteins, and to betaine, a process important in methyl-group metabolism [2, 3]. Betaine is an osmolyte and methyl donor that can serve as methyl group donor for conversion of homocysteine to form methionine [4]. Both choline and betaine are found in a wide variety of foods. The main food sources for choline are eggs, red meat, liver, seafood, and milk, whereas betaine is obtained mainly from grains, cereal, beets and spinach [1, 3, 5]. Growing evidence suggests that choline and betaine are involved in the pathogenesis of a variety of disease conditions, including the metabolic syndrome [6], fatty liver [7], cardiovascular diseases [8], and various cancers [1, 9].

Obesity is defined as an excess of body fat and is widely recognized as a chronic disease associated with many other more serious health problems including type 2 diabetes, cardiovascular disease, hypertension and at least a dozen of cancers [10]. The beneficial effect of choline and betaine on reducing body fat has long been studied in animals, such as rodents, pigs and chickens [11–16]. However, the data on humans are rare. One small study reported that, 11 of 23 lean males receiving a 2.5 g/day betaine supplementary with a simultaneous 6-week progressive resistance training program significantly increased their lean mass and reduced fat mass compared with non-betaine treatment placebo subjects [17]. In contrast, no change of body composition was found in a study examining 42 obese subjects after 12 weeks of 6 g/day betaine supplementation [18], neither in a study involving 34 young males after 10 days of 2 g/day betaine supplementation [19]. A cross sectional study in middle aged and elderly Norwegian population of both men and women showed that plasma betaine was inversely associated with obesity, while plasma choline showed a positive association with obesity [6]. The only obesity indexes used in this study were body mass index (BMI), body fat percentage and waist circumference (WC) and blood samples for choline and betaine measurements were from subjects that were non-fasting, which could cause great fluctuation in serum metabolites [20, 21]. Similarly, a recent study in middle aged and elderly Chinese found higher serum concentration of betaine was associated with better profiles of body composition, while serum choline levels only negatively correlated with BMI and weight [22]. This study was also complicated by examining only middle aged and elderly adults. Moreover, the variables related to body composition and serum choline and betaine levels were determined in different time points with a 3.2 years gap. Nevertheless, these two studies showed inconsistent results concerning the association of serum choline with obesity indexes. A larger study with large sample size, examining a greater variety of adult age groups and greater standardization of blood collection and measurement of anthropomorphic data, is needed to provide a clearer picture of the relationship between serum choline and betaine levels and body composition in the general adult population.

To the best of our knowledge, there is no previous study examining the effects of serum choline and betaine levels on body composition and fat distribution in North America and also in general aged adults. The current cross-sectional study was designed to investigate the association of serum choline and betaine levels with body composition in the general adult Newfoundland population after controlling for major potential confounding factors.

## Materials and methods

### Study population

A total of 1081 participants from the CODING (Complex Diseases in the Newfoundland population: Environment and Genetics) study were examined [23–25]. Inclusion criteria were as follows: 1) ≥19 years of age; 2) at least a third generation Newfoundlander; and 3) without serious metabolic, cardiovascular, or endocrine diseases.

Participants provided written and informed consent and the study received ethical approval from the Health Research Ethics Authority (HREA), Memorial University, St. John’s, Newfoundland, Canada, with Project Identification Code #10.33 (latest date of approval: 11 February 2016).

### Anthropometric and body composition measurements

Anthropometrics, body composition measurements were collected following a 12-hour fast. After urinating to empty their bladders, subjects were weighted to the nearest 0.1 kg in standard hospital gowns using a platform manual scale balance (Health O Meter, Bridgeview, IL). Standing height was measured using a fixed stadiometer to the nearest 0.1 cm. BMI (kg/m^2^) was calculated from weight and height in kilograms per square meter. WC was measured midway between the iliac crest and the lower rib, and hip circumference by the widest point over the buttocks below the iliac crest. Waist-to-hip ratio (WHR) was division of WC by hip circumference.

Dual Energy X-Ray Absorptiometry (DXA; Lunar Prodigy; GE Medical Systems, Madison, WI) was used for the measurement of percent trunk fat (TF%), percent android fat (AF%) and percent gynoid fat (GF%), total percent body fat (BF%) and total percent lean mass (LM%). The Lunar Prodigy software system determines regions automatically. Trunk fat region extends from the top of the shoulders to the top of the iliac crest, while the android fat region extends from the top of the second lumbar vertebra to the top of the iliac crest and the gynoid fat region extends down iliac crest twice the height of the android area. The enCORE (Ver 12.2, 2008, GE Medical Systems, Madison, WI) software package was used for DXA data acquisition. Visceral adipose tissue (VAT) content were estimated by CoreScan [26, 27] within the android region and percent visceral fat (VF%) were determined. Daily quality assurance was performed on the DXA scanner and the typical CV during the study period was 1.3% [23, 24].

### Lifestyle and dietary assessment

Information regarding participants’ lifestyles was collected through a self-administered screening questionnaire. The questions were related to demographics (age, gender and family origin), disease status, smoking status (yes/no), alcohol consumption (yes/no) and medicine use (yes/no). Women completed an additional questionnaire regarding their menopausal status. Physical activity patterns were measured using the ARIC Baecke Questionnaire, including Work Index, Sports Index, and Leisure Time Activity Index [28].

Dietary intake of each participant was assessed using a 124 item semi-quantitative Willett Food Frequency Questionnaire (FFQ) [29, 30]. The Willett FFQ obtains from subjects, the number of weekly servings of common food items consumed over the last year. Daily intake for each food item consumed was entered into the NutriBase Clinical Nutrition Manager (version 8.2.0; CybersoftInc, Phoenix, AZ) software package, and the total daily intake of calorie (kcal/day) for each individual were computed automatically [31, 32].

### Biochemical measurements

Venous blood samples were collected from all participants after a 12 hour fasting period. Serum samples were prepared and stored at −80°C for subsequent analysis. Serum concentrations of glucose, triacylglycerols (TG), total cholesterol (TC), and HDL cholesterol were measured on an Lx20 analyzer (Beckman Coulter Inc., Fullerton, CA) using Synchron reagents.

### Serum choline and betaine levels measurements

Liquid chromatography with tandem mass spectrometry (LC/MS/MS) was used for the quantification of serum choline and betaine levels [33, 34]. Serum proteins were precipitated with four volumes of ice-cold acetonitrile containing internal standards (d9-choline and d11-betaine) and 1μL supernatants were injected into a normal-phase HPLC column (Atlantis HILIC Silica 3 μM, 2.1x100 mm, Waters Corporation, Milford, MA). Choline and betaine compounds were eluted in an isocratic solvent system consisting of ammonium formate (15 mM, pH 3.0; 25%) and acetonitrile (75%) for 6 minutes at 0.6 mL/min with the HPLC (Waters Alliance 2795, Waters Corporation, Milford, MA). The column effluent was delivered to the tandem mass spectrometer (Micromass Ultima Triple-Quad MS, Waters Corporation, Milford, MA). The compounds were detected in positive multiple- reaction monitoring mode using the following m/z transitions: d9-choline 113>69, choline 104>60, d11-betaine 129>68, betaine 118>59. Different concentrations of mixed standards were used to prepare the calibration curves. Quality control samples were included in each batch.

### Data analysis

All data are presented as means ± standard deviation (SD). Serum glucose, serum TG and total calorie intake were log-transformed to normalize the data distributions to perform effective statistical analysis. Differences in anthropometrics, body compositions, total calorie intake, physical activity and serum measurements between males and females were assessed with independent Student’s *t* test.

Potential confounding factors were studied by investigating the correlation with body composition and serum choline or betaine levels with Pearson correlation analysis. Statistical interaction between serum choline, betaine levels and gender, medicine status, smoking status and alcohol status on the main outcomes was tested by analysis of covariance (ANCOVA). Partial correlation analysis controlling for age, total calorie intake, and physical activity level was used to evaluate the correlations of serum choline, betaine levels with weight, BMI, WC, WHR, AF%, GF%, VF%, TF%, BF% and LM% within male and female groups and also within medicine user and no-medicine user participants.

According to the criteria recommended by World Health Organization (WHO) [35], obesity status was categorized based on BMI into normal weight (18.5–24.9 kg/m^2^), overweight (25.0–29.9 kg/m^2^) and obese groups (≥30.0 kg/m^2^) in males. As the number of underweight subjects (BMI<18.5 kg/m^2^) was too small (n = 2) to perform any effective statistical analysis, they were excluded from analyses. Obesity status was also categorized by percent body fat according to age and gender specific criteria recommended by Bray [36]. Subjects less than 20 years old were excluded (n = 15) due to unavailability of criteria for this age group by Bray criteria. Serum choline and betaine levels were compared among adiposity groups by ANCOVA controlling for age, total calorie intake and physical activity level. In order to further explore the relationship between serum choline, betaine levels and obesity phenotypes, participants were divided into tertiles (low, medium, or high) based upon serum choline or betaine levels. Differences of obesity measurements among the three groups of different serum choline or betaine levels were assessed using ANCOVA controlling for age, total calorie intake and physical activity level.

All statistical analyses were performed using SPSS 20.0 (SPSS Inc., Chicago, IL). All tests were two sided and a p<0.05 was considered to be statistically significant.

## Results

### Physical parameters and serum choline, betaine levels

Demographic, physical and biochemical characteristics as well as serum choline, betaine levels of the participants are presented in Table 1. Female subjects were on average 2.7 years older than male subjects. Weight, Height, BMI, WC, WHR, android fat mass, VF%, visceral fat mass, LM%, total lean mass, physical activity and dietary calorie were significantly higher in males than females (p<0.001). AF%, GF%, gynoid fat mass, TF%, BF% and total fat mass were significantly lower in males as compared to females (p<0.001). Male participants had higher fasting glucose and TG compared to females but lower HDL cholesterol (p<0.001). Serum choline level was significantly lower while serum betaine level was significantly higher in males compared to females (p<0.001). No significant difference was evident in Hip, trunk fat mass and serum TC cholesterol level between genders.

**Table 1:** Characteristics of the participants by gender ^1^.

Variables	Male	Female	P^3^
Number^2^	536	545	
Age	42.2±13.3	44.9+11.3	0.000
Weight(kg)	88.2±15.7	70.3+13.2	0.000
Height (cm)	176.51±6.15	162.30±5.81	0.000
BMI (kg/m^2^)	28.27±4.58	26.7+5.0	0.000
WC (cm)	99.45±12.64	91.25+13.59	0.000
Hip (cm)	101.28±9.94	102.53+11.42	0.055
WHR	0.98±0.06	0.89±0.07	0.000
AF%	37.92±10.53	45.1±10.1	0.000
Android fat mass (g)	2740.95±1347.60	2488.96±1178.87	0.001
GF (%)	29.36±7.45	45.31±6.44	0.000
Gynoid fat mass (g)	4017.17±1540.31	5217.74±1571.98	0.000
TF (%)	31.87±8.83	40.06±8.14	0.000
Trunk fat mass (g)	14882.92±6551.96	14298.81±5628.56	0.116
VF (%)	1.47±0.90	0.99±0.67	0.000
Visceral fat mass (g)	1369.22±969.51	731.10±566.03	0.000
BF (%)	26.41±7.47	38.49±7.11	0.000
Total fat mass	24081.97±10092.19	27372.78±9515.64	0.000
LM (%)	69.78±7.12	57.89±67.30	0.000
Total lean mass	60822.12±7916.59	39573.09±5201.14	0.000
Physical activity	8.37±1.56	7.99±1.49	0.000
Calorie intake (kcal/day)	2201.63±1021.79	1823.06±787.67	0.000
Fasting glucose (mmol/L)	5.32±0.67	5.10±0.67	0.000
Fasting TG (mmol/L)	1.48±0.99	1.17±0.68	0.000
Fasting TC (mmol/L)	5.08±1.08	5.16±0.97	0.199
Fasting HDL-C (mmol/L)	1.19±0.28	1.52±0.38	0.000
Serum choline (μmol/L)	13.16±3.21	13.84±2.97	0.000
Serum betaine (μmol/L)	37.59±11.32	31.11±11.76	0.000

WC, waist circumference; WHR, Waist-to-hip ratio; AF%, percent android fat; GF (%), percent gynoid fat; TF(%), percent trunk fat (%); VF (%), percent visceral fat; BF (%), total percent body fat; LM (%), total percent lean mass.

^1^ All values are mean ± SDs;

^2^ Sample size range in each study group;

^3^ Significant differences between female and male group, based on independence sample Student’s t-test, Statistical significance was set to P<0.05.

### Correlation between serum choline, betaine levels and obesity measurements by gender

The correlations between serum choline, betaine levels and obesity measurements in different gender groups after adjusting for age, total calorie intake, and physical activity level are presented in Table 2. In male subjects, serum choline level was negatively correlated with weight, BMI, WC, WHR, TF%, BF% (r range from -0.111 to -0.185, p<0.05) and positively correlated with LM% (r = 0.116, p<0.01). In females, serum choline level was positively correlated with weight, BMI and WC (r range from 0.095 to 0.099, p<0.05).

**Table 2:** Partial correlations between serum choline and betaine levels with body composition^1^.

	Serum choline (μmol/L)		Serum betaine (μmol/L)	
	Male	Female	Male	Female
	r^2^ (P^3^)	r^2^ (P^3^)	r^2^ (P^3^)	r^2^ (P^3^)
Weight (kg)	-0.163(0.000)	0.096(0.026)	-0.223(0.000)	-0.005(0.914)
BMI (kg/m^2^)	-0.142(0.001)	0.099(0.021)	-0.225(0.000)	0.004(0.918)
WC (cm)	-0.147(0.001)	0.095(0.027)	-0.227(0.000)	-0.028(0.520)
WHR	-0.185(0.000)	-0.007(0.870)	-0.197(0.0000	-0.041(0.344)
TF (%)	-0.139(0.001)	0.014(0.751)	-0.209(0.000)	-0.033(0.439)
AF (%)	-0.079(0.069)	-0.015(0.724)	-0.181(0.000)	-0.055(0.200)
GF (%)	-0.077(0.077)	0.028(0.521)	-0.161(0.000)	-0.002(0.958)
VF (%)	-0.008(0.851)	0.009(0.840)	-0.119(0.006)	-0.079(0.068)
BF (%)	-0.111(0.011)	0.047(0.277)	-0.184(0.000)	0.006(0.885)
LM (%)	0.116(0.007)	-0.039(0.361)	0.186(0.000)	-0.008(0.846)

WC, waist circumference; WHR, Waist-to-hip ratio; TF(%), percent trunk fat (%); AF%, percent android fat; GF (%), percent gynoid fat; VF (%), percent visceral fat; BF (%), total percent body fat; LM (%), total percent lean mass.

^1^Partial correlations between serum choline, betaine levels (μmol/L) and obesity related indexes were controlling for age, total calorie intake, physical activity level;

^2^r: partial correlation coefficient;

^3^Statistical significance was set to P<0.05.

Serum betaine level was negatively correlated with all obesity measures (r range from -0.119 to -0.227, p<0.01) and positively correlated with LM% in males (r = 0.186, p<0.001). No significantly correlation was found between serum betaine level and obesity measures in females.

### Correlation between serum choline and betaine levels, and obesity measurements in non-medicated and medicated males

We also sought to explore the relationship between serum choline and betaine levels, and use of medicine in male participants controlling age, total calorie intake, and physical activity level, as shown in Table 3. Serum choline level was negatively correlated with weight, BMI, WC, WHR, TF%, BF% (r range from -0.135 to -0.255, p<0.05) and positively correlated with LM% (r = 0.143, p<0.05) in non-medicated males. Serum betaine level was negatively correlated with all obesity measures (r range from -0.186 to -0.280, p<0.01) and positively correlated with LM% in males (r = 0.233, p<0.001). In medicated males, no significantly correlation was found between serum choline level and obesity measures, while serum betaine was negatively correlated with weight (r = 0.159, p<0.05) and BMI (-0.155, p<0.05).

**Table 3:** Partial correlations between serum choline, betaine and body composition variables in males based on medication status^1^.

Male	Serum Choline (μmol/L)		Serum Betaine (μmol/L)	
	Non-medicated (n = 320)	Medicated (n = 216)	Non-medicated (n = 320)	Medicated (n = 216)
	r^2^ (P^3^)	r^2^ (P^3^)	r^2^ (P^3^)	r^2^ (P^3^)
Weight (kg)	-0.173(0.002)	-0.135(0.050)	-0.280(0.000)	-0.159(0.021)
BMI (kg/m^2^)	-0.140(0.013)	-0.124(0.071)	-0.262(0.000)	-0.155(0.023)
WC (cm)	-0.167(0.003)	-0.109(0.114)	-0.259(0.000)	-0.160(0.099)
WHR	-0.255(0.000)	-0.078(0.259)	-0.238(0.000)	-0.118(0.087)
TF (%)	-0.166(0.003)	-0.099(0.152)	-0.255(0.000)	-0.128(0.062)
AF (%)	-0.110(0.052)	-0.031(0.654)	-0.234(0.000)	-0.086(0.209)
GF (%)	-0.063(0.265)	-0.091(0.186)	-0.186(0.001)	-0.108(0.118)
VF (%)	-0.094(0.097)	0.083(0.227)	-0.216(0.000)	-0.007(0.918)
BF (%)	-0.135 (0.016)	-0.078(0.261)	-0.231(0.000)	-0.108(0.118)
LM (%)	0.143 (0.011)	0.081(0.239)	0.233(0.000)	0.108(0.115)

WC, waist circumference; WHR, Waist-to-hip ratio; AF%, percent android fat; GF (%), percent gynoid fat; TF(%), percent trunk fat (%); VF (%), percent visceral fat; BF (%), total percent body fat; LM (%), total percent lean mass.

^1^Partial correlations between serum choline, betaine levels (μmol/L) and obesity related indexes were in non-medicated and medicated males controlling for age, total calorie intake, physical activity level;

^2^r: partial correlation coefficient;

^3^Statistical significance was set to P<0.05.

### Comparison of serum choline and betaine levels among different adiposity groups

To further investigate the correlation of serum choline and betaine levels with obesity, male subjects were divided into three adiposity groups: normal weight, overweight and obese groups according to BMI recommended by WHO and according to %BF by Bray criteria.

As shown in Table 4, according to BMI, serum choline and betaine levels were significantly lower as adiposity status increased in both medicated and non-medicated subjects (p<0.05) and most prominent for serum betaine in non-medicated subjects (p<0.001) after controlling age, total calorie intake, and physical activity level. When subjects were divided by BF%, serum choline and betaine were significantly lower as adiposity status increased after controlling age, total calorie intake, and physical activity level in non-medicated males only (p<0.05).

**Table 4:** Comparison of serum choline and betaine levels in different obesity status defined by BMI and BF% in males^1^.

Grouped according to BMI^3^	Normal weight	Overweight	Obese	P^5^
Non-medicated	84^2^	142^2^	93^2^	
Choline (μmol/L)	13.21±2.69	12.99±3.05	12.44±2.82	0.043
Betaine (μmol/L)	40.46±12.88	36.66±9.64	32.74±8.12	0.000
Medicated	442	952	762	
Choline (μmol/L)	13.99±3.23	14.03±3.87	12.70±3.32	0.044
Betaine (μmol/L)	40.41±10.39	40.70±11.93	35.87±12.72	0.049
Grouped according to BF^4^	Normal weight	Overweight	Obese	P^5^
Non-medicated	89^2^	85^2^	136^2^	
Choline (μmol/L)	13.21±2.38	13.13±3.05	12.51±3.02	0.023
Betaine (μmol/L)	40.99±12.73	34.95±8.83	34.55±8.92	0.000
Medicated	51^2^	69^2^	91^2^	
Choline (μmol/L)	13.88±3.31	13.67±3.48	13.31±3.89	0.721
Betaine (μmol/L)	41.25±10.22	39.51±12.28	37.24±13.02	0.284

^1^All values are mean ± SDs;

^2^ Sample size range in each study group;

^3^The following subdivision is used as normal-weight (18.5–24.9 kg/m^2^), overweight (25.0–29.9 kg/m^2^), obese class (≥30.0 kg/m^2^) according to criteria of the World Health Organization;

^4^Subgroup were created by percent of body fat according to the age and gender specific criteria recommended by Bray;

^5^Significant differences between intervention groups. Data were assessed with ANCOVA controlling for age, total caloric intake, physical activity. Statistical significance was set to P<0.05.

### Comparison of body composition in different serum choline and betaine levels groups

Subjects were divided into tertiles (low, medium, or high) according to serum choline and betaine levels. As shown in Table 5, analysis of ANCOVA showed a significant inverse and dose-dependent association between serum choline levels and weight, BMI, WC, WHR, TF%, AF%, and BF% in non-medicated males (p<0.05) after controlling for age, total calorie intake, and physical activity. LM% presented a significantly positive and dose dependent association with serum choline in non-medicated males (p<0.01). The mean differences between high serum choline level and low serum choline level in non-medicated males were -6.80kg (weight), -1.56kg/m^2^ (BMI), -4.38cm (WC), -0.04 (WHR), and -3.56% (TF%), -2.89% (AF%), -2.84% (BF%) and 2.86% (LM%). No significant association was found between serum choline level and obesity measurements in medicated males.

**Table 5:** Comparison of obesity indexes according to serum choline levels in men^1^.

Choline (μmol/L)	Low^2^	Medium^2^	High^2^	P^4^
No-medicated^3^	106	107	107	
Choline (μmol/L)	9.87±1.39	12.77±0.64	15.98±2.01	0.000
	(5.59~11.64)	(11.65~13.89)	(13.90~22.68)	
Age (year)	36.79±11.38	37.46±113.30	40.35±13.30	-^5^
Calorie intake (kcal/day)	2254.85±822.98	2204.49±953.25	2335.53±1077.69	-^5^
Physical activity	8.51±1.35	8.34±1.39	8.74±1.74	-^5^
Weight (kg)	92.2-±15.06	85.09±15.03	85.40±14.11	0.000
BMI (kg/m^2^)	28.93±4.23	27.29±4.44	27.37±4.15	0.002
WC (cm)	101.48±12.01	95.86±12.31	97.10±12.04	0.000
WHR	1.00±0.05	0.96±0.06	0.96±0.05	0.000
TF (%)	33.07±9.04	30.41±9.00	29.51±8.78	0.001
AF (%)	38.55±10.32	36.24±10.68	35.66±11.16	0.028
GF (%)	30.44±7.39	29.45±7.38	28.35±7.62	0.208
VF (%)	1.25±0.73	1.26±0.92	1.26±0.84	0.464
BF (%)	27.44±7.71	25.49±7.46	24.60±7.19	0.008
LM (%)	68.67±7.40	70.63±7.13	71.53±6.86	0.005
Medicated ^3^	72	72	72	
Choline (μmol/L)	9.81±1.25	13.24±1.06	17.58±2.38	0.000
	(5.01~11.39)	(11.40~14.95)	(14.96~26.32)	
Age (year)	45.30±10.27	46.05±12.35	52.87±11.04	-^5^
Calorie intake (kcal/day)	2183.65±1236.83	2176.80±998.24	1960.85±1085.80	-^5^
Physical activity	8.10±1.71	8.31±1.48	8.04±1.66	-^5^
Weight (kg)	93.42±18.67	87.77±16.60	86.56±13.32	0.064
BMI (kg/m^2^)	29.92±5.38	28.52±4.90	28.28±4.14	0.113
WC (cm)	103.54±14.36	99.84±14.36	100.99±11.27	0.156
WHR	0.99±0.06	0.98±0.06	0.99±0.05	0.300
TF (%)	33.87±9.45	32.94±7.65	32.76±8.03	0.201
AF (%)	39.22±10.87	40.04±9.28	39.52±9.81	0.369
GF (%)	29.78±7.51	29.68±7.28	28.42±7.48	0.334
VF (%)	1.68±0.91	1.72±0.87	1.99±0.89	0.706
BF (%)	27.78±8.26	27.09±6.59	26.96±7.13	0.390
LM (%)	68.50±7.87	69.23±6.29	69.34±6.73	0.368

WC, waist circumference; WHR, Waist-to-hip ratio; AF%, percent android fat; GF (%), percent gynoid fat; TF (%), percent trunk fat (%); VF (%), percent visceral fat; BF (%), total percent body fat; LM (%), total percent lean mass.

^1^All values are mean ± SDs. BMI, body mass index; WC, waist circumference; WHR, waist-to-hip ratio;

^2^The subjects were divided to low, medium and high serum choline groups based on serum choline levels (umol/L).

^3^Sample size range in each study group;

^4^Significant differences between intervention groups. Data were assessed with ANCOVA controlling for age, total caloric intake, physical activity level. Statistical significance was set to p<0.05;

^5^As controlling factor, their differences were not analyzed.

As shown in Table 6, serum betaine was significant inversely correlated with all obese measurements (p<0.01) in non-medicated males. The mean differences between high serum betaine level and low serum betaine level in non-medicated males were -9.68kg (weight), -2.83kg/m^2^ (BMI), -8.13cm (WC), -0.03 (WHR), and -5.3% (TF%), -6.12% (AF%), -4.10% (BF%) and 3.59% (LM%). In medicated males, the significant dose-response association was found between serum betaine and weight, BMI, WC, TF%, GF%, BF% and LM% (p<0.05). The mean differences between high serum betaine level and low serum betaine level in non-medicated males were -10.26kg (weight), -2.92kg/m^2^ (BMI), -6.78cm (WC), -3.34% (%TF), -3.02% (GF%), -3.14% (BF%) and 2.98% (LM%).

**Table 6:** Comparison of obesity indexes according to serum betaine levels in men^1^.

Betaine (μmol/L)	Low^2^	Medium^2^	High^2^	P^4^
No-Medicated^3^	106	107	107	
Betaine (μmol/L)	26.27±3.26	34.97±2.54	48.41±8.61	0.000
	(14.97~30.79)	(30.80~39.48)	(39.49~83.32)	
Age (year)	40.00±13.56	39.40±12.79	35.23±11.40	-^5^
Calorie intake (kcal/day)	2145.89±874.62	2190.32±943.47	2457.64±1022.10	-^5^
Physical activity	8.35±1.39	8.62±1.55	8.62±1.57	-^5^
Weight (kg)	91.91±16.29	88.55±15.19	82.23±11.83	0.000
BMI (kg/m^2^)	29.09±4.63	28.24±4.16	26.26±3.70	0.000
WC (cm)	101.90±12.84	98.76±12.46	93.77±10.22	0.000
WHR	0.99±0.05	0.98±0.06	0.96±0.06	0.002
TF (%)	33.41±8.44	31.47±8.05	28.11±9.81	0.002
AF (%)	39.71±10.22	37.16±9.36	33.59±11.78	0.005
GF (%)	30.75±7.71	30.05±7.37	27.44±7.04	0.007
VF (%)	1.49±0.85	1.28±0.78	1.00±0.79	0.005
BF (%)	27.63±7.20	26.37±7.11	23.53±7.72	0.003
LM (%)	68.55±6.87	69.78±6.76	72.50±7.46	0.002
Medicated^3^	72	72	72	
Betaine (μmol/L)	27.54±3.96	36.97±2.72	52.69±9.72	0.000
	(14.98~32.68)	(32.69~42.63)	(42.64~83.09)	
Age (year)	44.93±11.88	48.16±11.02	51.30±11.48	-^5^
Calorie intake (kcal/day)	2262.34±1539.01	2075.62±806.38	1985.35±810.89	-^5^
Physical activity	8.04±1.71	8.23±1.48	8.15±1.67	-^5^
Weight (kg)	95.07±18.95	87.58±13.99	84.81±14.71	0.002
BMI (kg/m^2^)	30.70±5.35	28.17±4.32	27.78±4.39	0.001
WC (cm)	105.72±13.32	99.52±11.92	98.94±12.44	0.000
WHR	0.99±0.04	0.98±0.06	0.97±0.06	0.216
TF (%)	35.09±7.86	32.64±8.40	31.75±8.59	0.006
AF (%)	41.24±9.16	38.89±9.95	38.55±10.63	0.055
GF (%)	31.38±6.82	28.13±6.28	28.36±8.54	0.017
VF (%)	1.80±0.90	1.76±0.89	1.82±0.89	0.582
BF (%)	29.21±6.91	26.49±7.05	26.07±7.67	0.005
LM (%)	67.23±6.56	69.69±6.76	70.21±7.27	0.006

WC, waist circumference; WHR, Waist-to-hip ratio; AF%, percent android fat; GF (%), percent gynoid fat; TF(%), percent trunk fat (%); VF (%), percent visceral fat; BF (%), total percent body fat; LM (%), total percent lean mass.

^1^All values are mean ± SDs;

^2^The subjects were divided to low, medium and high serum choline groups based on serum choline levels (umol/L);

^3^Sample size range in each study group;

^4^Significant differences between intervention groups. Data were assessed with ANCOVA controlling for age, total calorie intake, physical activity level. Statistical significance was set to p<0.05;

^5^As controlling factor, their differences were not analyzed.

## Discussion

To the best of our knowledge, this is the first large cross-sectional study specifically designed to investigate the association between serum choline and betaine levels with DXA measured body composition in North America. The most important finding in the present study is that we found significant associations of higher serum choline and betaine levels with better body composition profile (lower %BF, higher %LM) in men of Newfoundland, but not in women. These beneficial associations are more pronounced in non-medicated men. Furthermore, the inverse correlation of serum betaine levels were with obesity measures was stronger than that of choline.

The strength of the current study lies in two aspects: 1) accurate measurement of physiological and biological markers, and 2) the systematic control of major confounding factors. It is critical to precisely quantify adiposity when study the influence of any factor on obesity status. Body composition in all subjects in the present study was measured using the same DXA system that produces accurate measurement of adipose tissue in the body with a low margin of error and is considered to be one of the most reliable measurements of adiposity [24]. Moreover, it is essential to identify major confounding factors in a large population based study and properly control these factors to obtain reliable results. In this study, numerous confounding factors that can potentially affect the relationship of the major factors under investigation has been identified and controlled. Obesity is a complex pathophysiological condition with numerous factors involved in its development [37]. Age and sex are primary important factors that affect the development of obesity [38]. Total dietary calorie intake and physical activity level are critical factor in maintaining balance of energy gain and expand that contribute to weight gain or loss [39]. In the present study, all of these confounding factors have been properly adjusted in all the analyses.

The first important finding in our study is that the beneficial association is gender specific. Negative association between serum choline and betaine levels with body fat percentage were only found in males. However, serum choline level was found positively correlated with weight, BMI and WC in females. The mechanism for the gender difference in this study is not clear. Numerous studies have proved that males and females are different in body composition, that is at least partly due to different effects of sex hormones [40, 41]. Women on average have 10–12% higher percentage of body fat than men and women store more fat in the gluteal-femoral region, whereas men store more fat in the visceral (abdominal) depot [24, 40, 41], which were consistent with our results. The metabolism of dietary nutrients is also different between males and females [42, 43]. A study in North Carolina reported a different dietary requirement for choline between men and women, which indicated the metabolism of choline may be different between men and women [44]. The different metabolic characteristics between men and women may be one of the potential reasons for the gender difference. Further studies are warranted to explore the exact mechanism. We also separated females based on menopause status, as menopause is usually accompanied by dramatic changes in sex hormones that can predispose women to weight gain [45]. No significant associations between serum betaine levels and body composition were found in both pre- and post-menopausal women, while the positive association between serum choline with weight, BMI and WC remained in post-menopausal women, but not pre-menopausal women (S1 Table). The other important finding in this study is that the associations between serum choline, betaine levels and body composition in males are profound in non-medicated than medicated men. Medication status is another important confounding factor [46], which can either impact weight gain or loss [47,48] and also can influence the nutrients metabolism and the subsequently serum metabolites profile [49]. We also found significant differences of serum choline and betaine levels between medicated and non-medicated males (S2 Table), which may be the consequence of medicine use. With dividing of the males to medication users and non-medication users, we found the significant correlations between serum choline and betaine levels with favorable body composition in males are more pronounced in non-medication users.

Our findings were partly supported by the two existing cross-sectional studies performed by Konstantinova *et al* and Chen *et al* in middle age and elder participants [6, 22]. Both of them reported a negative association of blood betaine levels with body fat in males. A positive relationship between plasma choline with BMI and WC was also reported in females by Konstantinova *et al* [6]. These results are consistent with our findings. However, both of them also reported a significant negative correlation between serum betaine level and body fat in females, which was weaker than in males. Furthermore, Konstantinova *et al* did not find any significant associations for serum choline with BMI or percent body fat in males [6]. While, Chen *et al* reported a significantly negative association of serum choline with weight and BMI, but not body composition in women and men. Moreover, none of the two studies considered medication use [6, 22]. The inconsistent results may come from the differences of population, time of collect samples, and range of ages, *et al* [50, 51].

Some interventional studies in both humans and animals can also support our findings. To date, only three interventional studies in humans have been performed to examine the effects of betaine supplementary on body fat. A significant decrease in fat mass and percent body fat was observed among 11 male participants undergoing a periodical training program [17], while two other studies failed to replicate the results [18,19]. No data is available regarding the effects of choline supplementation in humans. Animal studies have provided the main evidence of the beneficial effect of choline and betaine on body composition. Choline or betaine supplementation improves the growth performance and carcass characteristics of gilts, other pigs, meat ducks, chickens and rodents [11–16]. Our previous study has also reported a significant relationship between higher dietary choline, betaine intakes and favorable body composition in Newfoundland population [52]. Our current study fills the gap of the correlation between serum choline and betaine levels with body composition in Newfoundland population.

The exact mechanisms by which choline and betaine improve body composition are unclear, while several mechanisms may explain the association. Choline or betaine supplementation has been found to promote fatty acid β-oxidation in pigs and humans by enhancing muscle carnitine accretion and thereby increase carnitine palmitoyl transferase I-mediated free fatty acid translocation into the mitochondria [15, 53, 54]. Betaine supplementation can decrease the capacity for fatty acid and triglyceride synthesis by decreasing the activity of acetyl-CoA carboxylase, fatty acid synthase and malic enzyme and their mRNA expression in adipose tissue [15, 55]. Betaine reduces uptake of triglycerides from circulating lipoproteins in broilers by decreasing the mRNA expression of lipoprotein lipase [55]. Betaine-related methylation of homocysteine to methionine may reduce the substrate (acetyl-coenzyme A) for fatty acid synthesis [54]. Dietary betaine mobilizes fat degradation in adipose tissue of pigs by increasing hormone sensitive lipase activity [56]. Betaine supplementation promotes protein synthesis by stimulating growth hormone secretion and improving insulin and Insulin-like Growth Factor 1 receptor signalling [57, 58]. Accumulation of betaine in cells increases sarcoplasmic osmolality which may also contribute to the increase of muscle mass [59, 60].

A number of limitations should be considered in this study. First, this is a cross-sectional study, which could not establish a cause-effect relationship. A further longitudinal study is warranted to fill the knowledge gap. Secondly, although multiple factors, including socio-demographic characteristics, lifestyle habits, physical activities and calorie intake were comprehensively adjusted in our analysis, residual confounding factors, including genetics and unknown or poorly measured factors could not be completely ruled out.

## Conclusions

In conclusion, the present study provides evidence for the first time, in the large Newfoundland population, that higher serum choline and betaine levels were associated with a more favorable body composition (lower body fat and higher lean body mass) in males. In addition, this favorable association was independent of age, total calorie intake, physical activity level, while more pronounced in non-medication users. The beneficial correlations for serum betaine were stronger than choline.

## Supporting information

S1 Table Partial correlations between serum choline, betaine and body composition variables in females based on menopausal status. (DOC)

S2 Table Characteristics of the participants based on medication status. (DOC)
