# Supplementary material for: Higher serum choline and betaine levels are associated with better body composition in male but not female population
Source: PLoS One. 2018 Feb 20;13(2):e0193114. doi: 10.1371/journal.pone.0193114 (PMC5819804; doi:10.1371/journal.pone.0193114)
Supplement: S1 Table — (DOC) [file pone.0193114.s001.doc]

**S1 Table. Partial correlations between serum choline, betaine and body composition variables in females based on menopausal status**

|  | Serum Choline (umol/L) | | |  | Serum Betaine (umol/L) | | |
| --- | --- | --- | --- | --- | --- | --- | --- |
|  | Pre-menopausal (n^1^=324) |  | Post- menopausal (n^1^=221) |  | Pre-menopausal (n^1^=324) |  | Post- menopausal (n^1^=221) |
|  | r^2^ (P^3^) |  | r^2^ (P^3^) |  | r^2^ (P^3^) |  | r^2^ (P^3^) |
| Weight (kg) | 0.040(0.477) |  | 0.203(0.003) |  | -0.054(0.336) |  | 0.042(0.539) |
| BMI (kg/m2) | 0.050(0.373) |  | 0.203(0.003) |  | -0.037(0.515) |  | 0.040(0.559) |
| WC (cm) | 0.057(0.310) |  | 0.184(0.006) |  | -0.055(0.331) |  | -0.014(0.842) |
| WHR | -0.012(0.832) |  | -0.003(0.960) |  | -0.036(0.519) |  | -0.051(0.453) |
| TF (%) | -0.001(0.981) |  | 0.085(0.211) |  | -0.011(0.849) |  | -0.073(0.287) |
| Trunk fat mass(g) | 0.017(0.767) |  | 0.163(0.016) |  | -0.059(0.291) |  | -0.034(0.623) |
| AF (%) | -0.015(0.794) |  | 0.022(0.749) |  | -0.026(0.650) |  | -0.118(0.083) |
| Android fat mass (g) | 0.039(0.481) |  | 0.115(0.090) |  | -0.057(0.312) |  | -0.088(0.194) |
| GF (%) | 0.042(0.450) |  | 0.048(0.478) |  | -0.030(0.597) |  | 0.025(0.710) |
| Gynoid fat mass(g) | 0.055(0.330) |  | 0.153(0.023) |  | -0.035(0.528) |  | 0.062(0.360) |
| VF (%) | 0.022(0.700) |  | 0.007(0.920) |  | -0.016(0.769) |  | -0.175(0.010) |
| Visceral fat mass | 0.045 (0.422) |  | 0.086(0.204) |  | -0.037(0.505) |  | -0.129(0.058) |
| BF (%) | 0.029 (0.604) |  | 0.127(0.061) |  | 0.008(0.888) |  | 0.001(0.987) |
| Total body fat mass (g) | 0.045 (0.424) |  | 0.200(0.003) |  | -0.029(0.602) |  | 0.034(0.621) |
| LM (%) | -0.021 (0.714) |  | -0.122(0.073) |  | -0.011(0.839) |  | -0.001(0.990) |

WC, waist circumference; WHR, Waist-to-hip ratio; AF%, percent android fat; GF (%), percent gynoid fat; TF(%), percent trunk fat (%); VF (%), percent visceral fat; BF (%), total percent body fat; LM (%), total percent lean mass.

^1^Sample size range in each study group；

^2^controlled for age, total calorie intake, physical activity；

^3^Statistical significance was set to P<0.05.
