# Supplementary material for: Higher serum choline and betaine levels are associated with better body composition in male but not female population
Source: PLoS One. 2018 Feb 20;13(2):e0193114. doi: 10.1371/journal.pone.0193114 (PMC5819804; doi:10.1371/journal.pone.0193114)
Supplement: S2 Table — (DOC) [file pone.0193114.s002.doc]

**S2 Table.** **Characteristics of the participants based on Medication status^1^**

| Variables | Male | | Female | |  |  |
| --- | --- | --- | --- | --- | --- | --- |
|  | Non-medicated | Medicated | Non-medicated | Medicated | P^1^ | P^2^ |
| Number^2^ | 320 | 216 | 220 | 325 |  |  |
| Age | 38.07±12.71 | 48.13±11.71 | 44.97±8.86 | 44.91±12.71 | 0.000 | 0.947 |
| Weight(kg) | 87.47±15.02 | 89.15±16.54 | 69.48±11.80 | 70.79±14.07 | 0.223 | 0.238 |
| Height (cm) | 177.14±6.04 | 175.57±6.22 | 162.24±5.36 | 162.33±6.03 | 0.004 | 0.854 |
| BMI | 27.84±4.31 | 28.88±4.86 | 26.42±4.47 | 26.88±5.25 | 0.009 | 0.275 |
| WC (cm) | 98.04±12.31 | 101.39±12.89 | 89.83±12.45 | 92.19±14.21 | 0.003 | 0.048 |
| Hip (cm) | 100.32±9.55 | 102.64±10.39 | 102.03±10.64 | 102.83±11.92 | 0.008 | 0.426 |
| WHR | 0.98±0.06 | 0.99±0.06 | 0.88±0.07 | 0.89±0.07 | 0.031 | 0.011 |
| AF (%) | 36.78±10.80 | 39.56±9.95 | 44.14±10.21 | 45.70±9.93 | 0.002 | 0.077 |
| Android fat mass (g) | 2555±1272 | 3006±1414 | 2347±1055 | 2580±1240 | 0.000 | 0.023 |
| GF (%) | 29.40±7.51 | 29.29±7.40 | 45.09±6.44 | 45.49±6.42 | 0.873 | 0.476 |
| Gynoid fat mass (g) | 4028±1542 | 3993±1543 | 5118±1418 | 5289±1667 | 0.796 | 0.217 |
| TF (%) | 30.95±9.06 | 33.16±8.37 | 39.35±8.19 | 40.54±8.00 | 0.004 | 0.092 |
| Trunk fat mass (g) | 14135±6298 | 15934±6795 | 13725±5169 | 14671±5872 | 0.002 | 0.055 |
| VF (%) | 1.25±0.83 | 1.79±0.89 | 0.91±0.59 | 1.04±0.72 | 0.000 | 0.013 |
| Visceral fat mass (g) | 1153±863 | 1680±1035 | 657±492 | 778±605 | 0.000 | 0.011 |
| BF (%) | 25.81±7.54 | 27.26±7.32 | 37.98±7.01 | 38.84±7.11 | 0.028 | 0.165 |
| Total fat mass | 23353±9913 | 25093±10305 | 26651±8751 | 27852±9958 | 0.051 | 0.150 |
| LM (%) | 70.31±7.22 | 69.04±6.96 | 58.33±6.66 | 57.58±6.72 | 0.044 | 0.204 |
| Total lean mass | 60812±7578 | 60782±8362 | 39489±4713 | 39606±5511 | 0.965 | 0.797 |
| PA | 8.54±1.51 | 8.14±1.62 | 8.10±1.38 | 7.91±1.56 | 0.004 | 0.133 |
| Calorie intake (kcal/day) | 2269±958 | 2107±1107 | 1818±712 | 1816±824 | 0.015 | 0.374 |
| Fasting glucose (mmol/L) | 5.16±0.45 | 5.55±0.84 | 5.06±0.69 | 5.04±0.65 | 0.000 | 0.713 |
| Fasting TG (mmol/L) | 1.40±0.91 | 1.59±1.10 | 1.04±0.66 | 1.25±0.69 | 0.035 | 0.001 |
| Fasting TC (mmol/L) | 5.14±1.09 | 5.00±1.03 | 5.17±0.98 | 5.17±0.97 | 0.008 | 0.983 |
| Fasting HDL-C (mmol/L) | 1.20±0.26 | 1.19±0.31 | 1.56±0.40 | 1.50±0.36 | 0.616 | 0.056 |
| Serum choline (µmol/L) | 12.89±2.90 | 13.56±3.59 | 13.37±2.85 | 14.17±3.01 | 0.024 | 0.002 |
| Serum betaine (µmol/L) | 36.54±10.57 | 39.07±12.12 | 32.88±10.55 | 29.89±12.28 | 0.011 | 0.003 |

WC, waist circumference; WHR, Waist-to-hip ratio; AF%, percent android fat; GF (%), percent gynoid fat; TF(%), percent trunk fat (%); VF (%), percent visceral fat; BF (%), total percent body fat; LM (%), total percent lean mass.

^1^All values are mean ± SDs；

^2^Sample size range in each study group；

P^1^ Significant differences between medicated and non-medicated male groups, based on independence sample [Student's t-test,](http://www.baidu.com/link?url=7vvNS9bE0p4C3xNhiR2C2XJpVuG1E6PRTbwAcQt5ut_08T_K_ROTKvknlovBkn77Q5pD8njwQlAjGMntgIU_R_) Statistical significance was set to p<0.05；

P^2^ Significant differences between medicated and non-medicated female groups, based on independence sample [Student's t-test,](http://www.baidu.com/link?url=7vvNS9bE0p4C3xNhiR2C2XJpVuG1E6PRTbwAcQt5ut_08T_K_ROTKvknlovBkn77Q5pD8njwQlAjGMntgIU_R_) Statistical significance was set to p<0.05.
